# Exploring medical students’ perceptions of family medicine in Kyrgyzstan: a mixed method study

**DOI:** 10.1186/s12909-023-04126-2

**Published:** 2023-04-12

**Authors:** Olivia Heller, Zhyldyz Ismailova, Damira Mambetalieva, Nurlan Brimkulov, David Beran, Mathieu Nendaz, Nu V. Vu, Louis Loutan, Anne Baroffio

**Affiliations:** 1 Division of Tropical and Humanitarian Medicine, Geneva University Hospitals, Rue Gabrielle-Perret-Gentil 6, Geneva, 1211 Switzerland; 2 Medical Education Reform (MERproject), Bishkek, Kyrgyzstan; 3 Kyrgyz State Medical Academy, Bishkek, Kyrgyzstan; 4 Unit of Development and Research in Medical Education, Faculty of Medicine, University of Geneva, Rue Michel Servet 1, Geneva, 1211 Switzerland

**Keywords:** Family Medicine, Developing country, Medical education, Mixed-methods, Qualitative research, Medical students, Primary care

## Abstract

**Background:**

Despite knowing that health systems with strong primary care improve overall health outcomes within a population, many countries are facing a global trend of declining interest and shortage of family doctors. This is the case of the Kyrgyz Republic, in which rural areas are struggling to attract and retain family medicine (FM) doctors. This study aims to explore how Kyrgyz medical students perceive FM and the factors that influence their specialty choice.

**Methods:**

This study used a cross-sectional explanatory sequential design, including quantitative survey and focus group discussions that were carried out at the Kyrgyz State Medical Academy (KSMA) in Bishkek in 2017. Overall, 66% (953 out of 1449) of medical undergraduate students registered in year 1, 4 and 6 completed the survey, and 42 participated in the focus groups. The results were organized around 7 factors influencing perceptions and attitudes towards FM identified through a qualitative systematic review.

**Results:**

The interest of Kyrgyz students for FM was the lowest of all specialties. Access to high medical technologies, career opportunities, salary, patient interaction and possibility to work abroad were the five most important factors influencing specialty choice. FM was perceived as a difficult profession, yet with poor prestige, insufficient remuneration, limited career possibilities and poor working conditions, especially in rural areas. The academic discourse, which disregards FM specialty had a negative influence on student’s perceptions and prevented students’ ability to identify with the practice of family medicine. However, students’ awareness of their social accountability arose as a positive leverage to increase the choice of FM, provided other problems were solved.

**Conclusion:**

This study highlighted key factors responsible for the low number of students choosing to become FM in Kyrgyzstan. The first major factor, presumably specific to many low- and lower-middle- income countries was the poor working conditions in remote areas. The second factor, common to many countries, was the distorted image of FM and its specialty transmitted through the medical schools’ institutional culture which does not value FM through positive role models. This study served as a basis to establish a strategy to promote FM within the KSMA and potentially at National level.

**Supplementary Information:**

The online version contains supplementary material available at 10.1186/s12909-023-04126-2.

## Background

Primary health care (PHC) was put forward 42 years ago with the Alma Ata declaration as a set of values, principles and approaches aimed at raising the level of health in disadvantaged populations.[1, 2] In 2018, the Astana declaration renewed these key principles as a driving force for achieving the Sustainable Development Goals (SDGs).[3] Evidence has shown that countries reorienting their health systems towards PHC are better placed to achieve the SDGs than those with hospital focused system.[4, 5] Developing stronger PHC with General Practitioners /Family Medicine doctors is linked to better outcomes, lower costs, and improved health equity.[6] For the purpose of this paper, family medicine (FM) doctors will be used as equivalent to general practitioner as this is the term commonly referred to in Kyrgyzstan.

Despite clear progress, the development of FM continues to face wide range of challenges.[7] The World Health Organization (WHO) alerts on the projected shortfall of 18 million health workers, primarily in low- and lower-middle- income countries (LMIC), by 2030.[8] This shortage will have serious implications for the health of billions of people across all regions of the world if not addressed.

The global human resource (HR) challenges described above echoes the situation in Kyrgyzstan a landlock country in Central Asia. In Kyrgyzstan, FM doctors represent about 16% of doctors corresponding to a medical density of 24.7/10,000 population, while WHO recommends 44.5/10,000. The current deficit especially impacts rural areas, where the few remaining FM doctors are either beyond or near retirement age.[8–10] Nevertheless, PHC remains the first point of entry into the healthcare system for most people in Kyrgyzstan. Besides the lack of FM already practicing in the health system, this specialty is not well-recognized and valued by the Kyrgyz population and is unpopular for medical students leading to very few young doctors deciding to follow this professional track. [11–13]

Since its independence from the former Soviet Union in 1991, Kyrgyzstan has embarked on a major healthcare reform, reducing the overall hospital capacity, moving towards more ambulatory care, retraining and developing a stronger PHC base with FM doctors. Since 2007, The Geneva University Hospitals (HUG) and the Unit of Development and Research in Medical Education (UDREM)[14] at the University of Geneva have been providing technical support for medical training through the Medical Education Reform (MER) project financed by the Swiss Agency for Development and Cooperation (SDC).[15] The main goals of this project are to improve the quality of the pre-graduate, post-graduate and continuing medical education program by strengthening the instructional and organizational aspects of the curriculum; introducing more interactive teaching methods and active clinical experiences and practice to students; improving the students’ assessment system leading to a national certification examination, and reinforcing the priority towards FM.

While global efforts to develop FM have been gaining momentum over the past decade with several studies undertaken in the high income countries [16–21]. Few analyses focus on the factors influencing medical students’ specialty choice in LMICs and perception of FM.[10, 22] This study aims to explore how Kyrgyz medical students perceive FM and the factors that may influence their choice of specialty.

## Methods

### Study design and setting

**Fig. 1:**
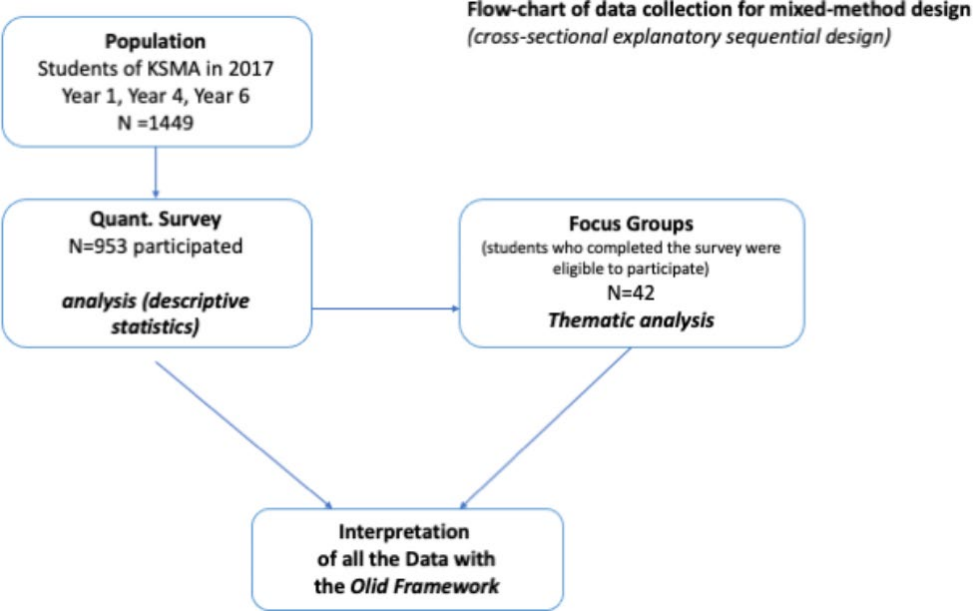
Flow-chart of data collection

This study is a cross-sectional explanatory sequential design, including quantitative survey and focus group discussions that were carried out at the Kyrgyz State Medical Academy (KSMA) in Bishkek in 2017. **(**Fig. 1**– flow chart)**.

### Context

**Fig. 2:**
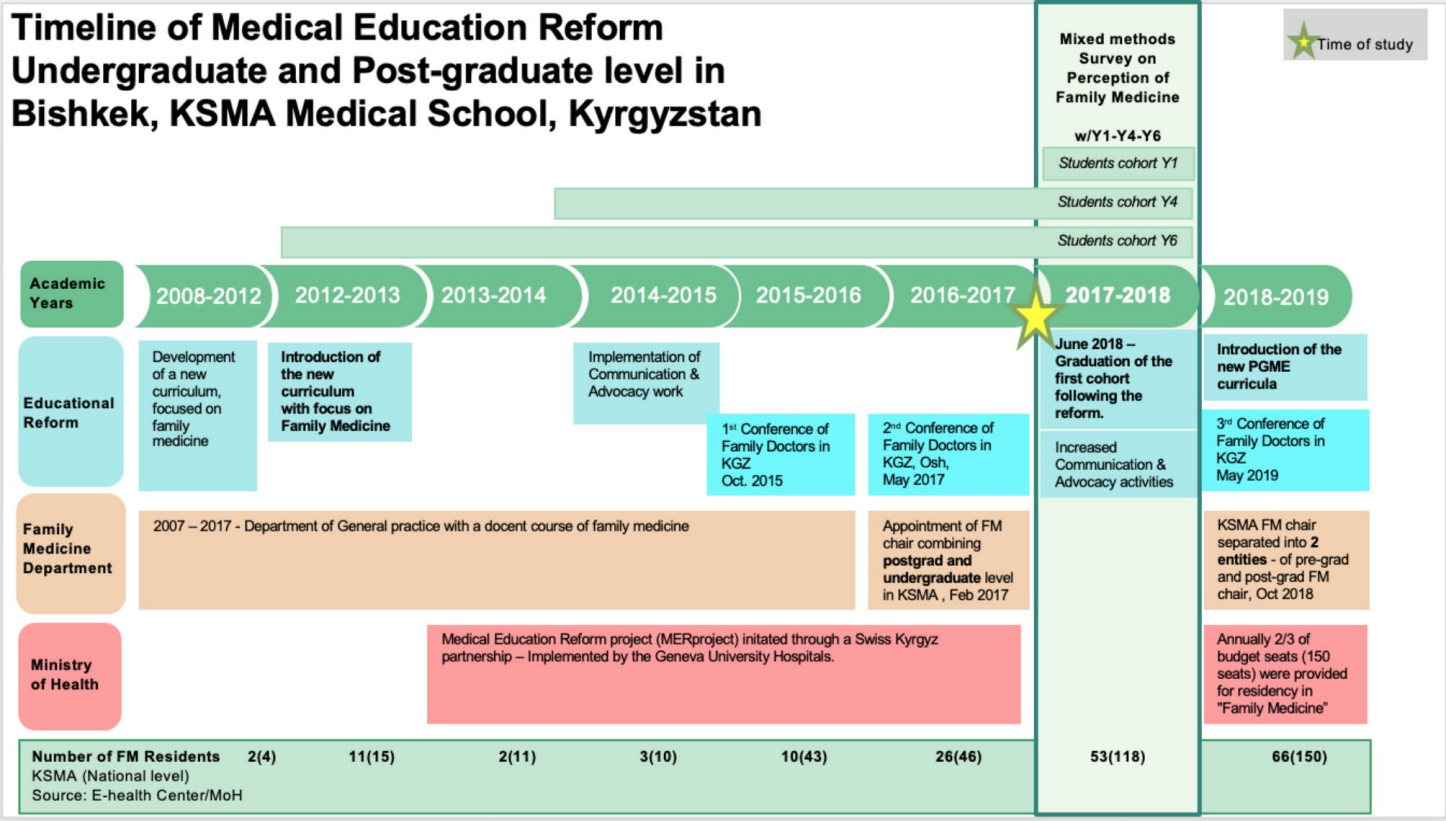
Timeline of Medical Education Reform in KSMA Medical School, Kyrgyzstan Description: The reform started to be implemented in September 2012 and the first graduates of this reform received the certificate in June 2018. The survey was conducted in autumn 2017, interviewing students from Y1, Y4, Y6, all having benefited from the reformed programme. Since 2017, the post-graduate training is under the responsibility of the Ministry of Health. Until that year it was under the responsibility of the Minister of Education and Medical Schools. This shift was an important milestone as it links the training content to health issues and not just education. Important efforts to promote family medicine through advocacy and communication strategies, video, leaflets, conference were implemented since 2014 in parallel to reforming the curriculum. The package of measures bared its fruits with number of residents increasing in the subsequent years, – 150 – choosing FM in 2019 with over 50% going to the regions.

The specific situation of Kyrgyzstan stemmed from a previous Soviet system which favoured specialties and sub-specialties.[10, 22] The medical education system is still very much influenced by specialists, with very little recognition of FM. The pre-graduate medical curriculum is exclusively taught by specialists, who do not have a clear idea of what FM is. At national level, in 2013, 15 residents opted for the FM speciality, versus 11 in 2014, 10 in 2015. (MER-Project Data). The promotion of FM and increase in the number of FM doctors has become a key priority for the Ministry of Health (MOH) and results started to show increase from 2016. (Fig. 2 - Timeline). Medical studies in Kyrgyzstan are 6 years long. The KSMA curriculum reform was initiated in 2012 and the first cohort of students to graduate with the revised program focusing on FM took place in June 2018. In parallel, the post-graduate training for FM specialty was reformed and a new two-year residency training was introduced in 2018 (instead of the initial one-year residency training), with 150 positions for FM available out of 894 residency positions throughout the country. The post-graduate training for the other specialties lasts at least three years.

### Participants

The target group were medical undergraduate students at KSMA registered at university in 2017/2018 in year 1, 4 and 6 representing a total cohort of 1449 students.

### Ethics approval

Prior to any data collection, the study was submitted in 2017 to the Ethics Committees in both Geneva and Bishkek (Commission Cantonale d’Ethique de la Recherche (CCER) in Geneva and the KSMA Ethical board in Bishkek), who designated the study as exempted from formal review.

### Instrument and data collection

The data collection tools (survey and interview guide for the focus group discussions) were translated into Russian and back-translated into English to ensure the translation’s quality (Additional file 1). A pilot test was carried out to estimate the duration of survey administration and to provide guidance for the data collection team in the planning. The data collection took place from June to December 2017. The Kyrgyz partners (ZI, DM, NB) with support from the Head of the Educational Department at KSMA took care of organization and data collection. Clearance from the administration of KSMA and the rector were obtained prior to the data collection. The first author went on site to moderate the focus group discussions in November 2017.

#### Quantitative survey

For maximum participation, national partners introduced the survey’s objectives to the students during a lecture preceding the survey. Students were invited to sign an informed consent form prior to survey’s administration, which took place during an assigned class on computers in a centre equipped with 65 computers. The survey was self-administered at three key training stages; (year 1, start of the pre-clinical teaching; year 4, between pre-clinical and clinical teaching; year 6, fully clinical teaching) and students took on average 20 min to fill them.

The survey was adapted from an existing survey used in the Republic of Tajikistan in 2014 for a similar project.[23] In order to enable comparison, the survey was only adapted when necessary to reflect the Kyrgyz context, such as name of curriculum and modules, and factors influencing specialty choice… The survey was divided into 6 sections: (1) Socio-demographic data (10 items) (2) Choice of specialty (7 items) and influencing factors (12 items) (3) Perception of FM specialty (11 items) and in comparison, with other specialties (6 items) (4) Type of comments about FM (5 items) (5) Perception of the quality and impact of the training in medical school on perception of FM (6) Perception of post-graduate training (3 items) given to year 6 students only.

#### Focus group discussion

The focus group participants were selected from the participants who responded to the survey in order to be as representative as possible of the whole student population with regards to their origin (urban or rural), gender and mode of financing their medical education (government subsidized^1^ or private students). Six focus groups (2 per study year) were organized with 5–9 students per group and consisted in in-depth discussions about 3 themes based on the preliminary results of phase 1: (a) the factors that influenced their specialty choice (students of year 6 only), (b) their views on specializing in family medicine in comparison with other specialties, (c) their views on the new curriculum and their recommendations.

To stimulate the discussion about the image of Family Medicine compared to other specialties, participants were asked to rank a set of 13 specialties^2^ including family medicine doctors according to their level of difficulty, attractiveness and prestige. This was done by sorting cards into piles. This approach allowed access to people’s perceptions and invites the participants to structure and justify their representations.[24]. Students organized the cards on a range of 4 to 10 levels and the level of ranking of FM was standardized on a maximum of 10 to allow comparison per year and per perception (for example if FM was classified on the 4th level out of 5, it was then standardized as 8 out of 10).

### Data management and analysis

Data from the questionnaires were imported into an excel spreadsheet to enable descriptive statistics to be drawn from students’ answers.

Focus group discussions were recorded, transcribed verbatim by the local investigator (ZI) and translated from Russian into English. The transcripts were analysed using the MAXQDA 2018[25] programme in a deductive approach using a framework based on 7 factors identified from a qualitative systematic review by Olid et al.[26] looking at students’ attitudes and perceptions towards FM. (Table 1)

**Table 1:** The seven factors identified by Olid et al.[22] and used as themes for the qualitative analysis of the focus groups

Factors influencing perceptions and attitudes towards FM	Description
1) broad scope and context of practice	*Perception of varied specialty; Broad practice; Holistic perspective; Continued and long-term care; Preventive and public health activities*
2) lower interest or intellectually less challenging	*Not intellectually challenging; Treating common diseases; Serious problems referred to specialists; Superficial, “mundane” and repetitive; Less action and less technology; Gatekeepers of health care system; Just triage patients*
3) influence of role models and society, other professionals and family	*Negative comments and attitudes from other specialist, teachers, residents, colleagues, peers influence students’ career interests; Students feel pressure from family, friends and society to choose a different specialty; Influence of role models on students’ perception; Negative media coverage impacts students’ perception*
4) lower prestige	*Lower status of FM compared to other specialties, professionally and socially; Choice of FM is an inferior and second choice*
5) low remuneration	*Poor remuneration compared to other specialties; Difficulty to generate an additional income in the private sector*
6) medical school influence	*Undergraduate experiences in FM influence career intentions; Exposure can be more or less stimulating than expected; If no exposure to FM, poor idea of what FM practice is; Length and quality of exposure influence specialty choice*
7) postgraduate training	*Less intensive and shorter training considered as a positive element; Flexibility, well-structured program and lack of competition are positive aspects*

The final analysis consisted in aligning findings from the survey and from the FGD in each of the 7 Olid et al.[26] themes. Findings were then synthetized and interpreted for each theme. In addition, new emerging themes were extracted and representative quotes were chosen for each theme. (Table 3) The label following the quotes indicates the number of the FG and the year of study.

## Results

### a. Participants

Overall, 66% of registered students (953 out of 1449) completed the survey, with a similar percentage of students per study year (Table 2). Out of the 953 surveyed students, 63% were female which is representative of the higher proportion of female student population at KSMA (60%). 63% came from cities and 24% from rural region. Overall, 47% of students received subsidized tuition from the government, the remaining 53% paid themselves for their studies. The proportion of subsidized students corresponds to the number of grants given by the government and this can vary from year to year. Forty-two students were recruited randomly for the focus group discussions, including 31 females, 20 government subsidized, and 32 coming from cities.

**Table 2:** Characteristics of survey and focus group discussion participants

Survey: General information about Participants									
Study year	Number of registered students		Number of completed survey		Female	Government subsidized			From urban areas
Year 1	400		270 (67.5%)		64%	60%			66%
Year 4	559		368 (65.8%)		61%	52%			63%
Year 6	490		315 (64.3%)		63%	33%			58%
**Total**	**1449**		**953 (66%)**		**598 (63%)**	**455 (47%)**			**597 (63%)**
**Focus Groups Discussions: General information about Participants**									
**Study year**		**Number**	**Female**	**Government Subsidized**			**City**	**Have visited FM**	
Year 1		12	11	7			9	9	
Year 4		17	10	8			13	6	
Year 6		13	10	5			10	6	
**Total**		**42**	**31**	**20**			**32**	**21**	

### b. Students’ interest for various specialties

**Fig. 3:**
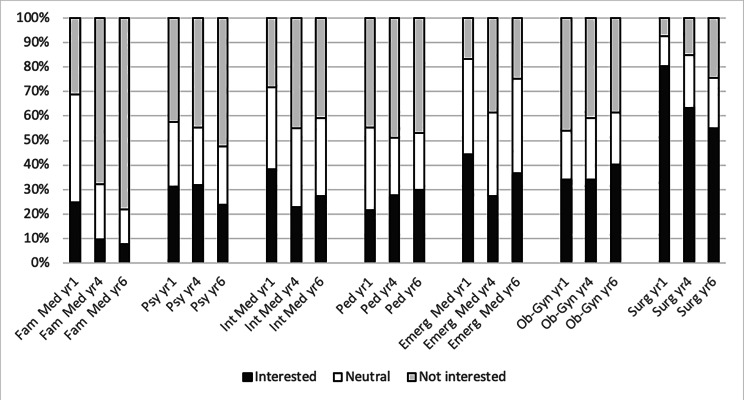
Percent of Kyrgyz medical students interested in working in different specialties at 3 different times of their medical training Legend to table: The vertical axis presents the percent of students interested, neutral or not interested in seven main specialties including FM/GP at year 1, 4 and 6 of their medical training. Total N = 953 students from KSMA (year 1: 270, 4: 368 and 6: 315). They were surveyed in 2017 and answered the question “How much are you interested in working in each of the following specialties/career options after your studies”. Fam Med = Family Medicine; Psy = Psychiatry; Int Med = Internal Medicine; Ped = Paediatric; Emerg Med = Emergency Medicine; Ob-Gyn = Obstetrics-Gynaecology; Surg = Surgery.

Figure 3 illustrates the percent of Kyrgyz medical students interested in working in different specialties at year 1, 4 and 6 of their medical training. The interest for FM was the lowest of all specialties and decreased over the study years (24%, 10% and 8% in year 1, 4 and 6 respectively). The highest interest was for surgical specialties, which also decreased over the study years, with more than 50% students still interested at year 6 (80%, 63% and 55% respectively). Finally, the interest in other specialties (psychiatry, internal medicine, paediatrics, emergency medicine, and obstetrics-gynaecology) ranged from 20 to 45% with no clear trend over the study years.

### c. Factors influencing specialty choice

**Fig. 4:**
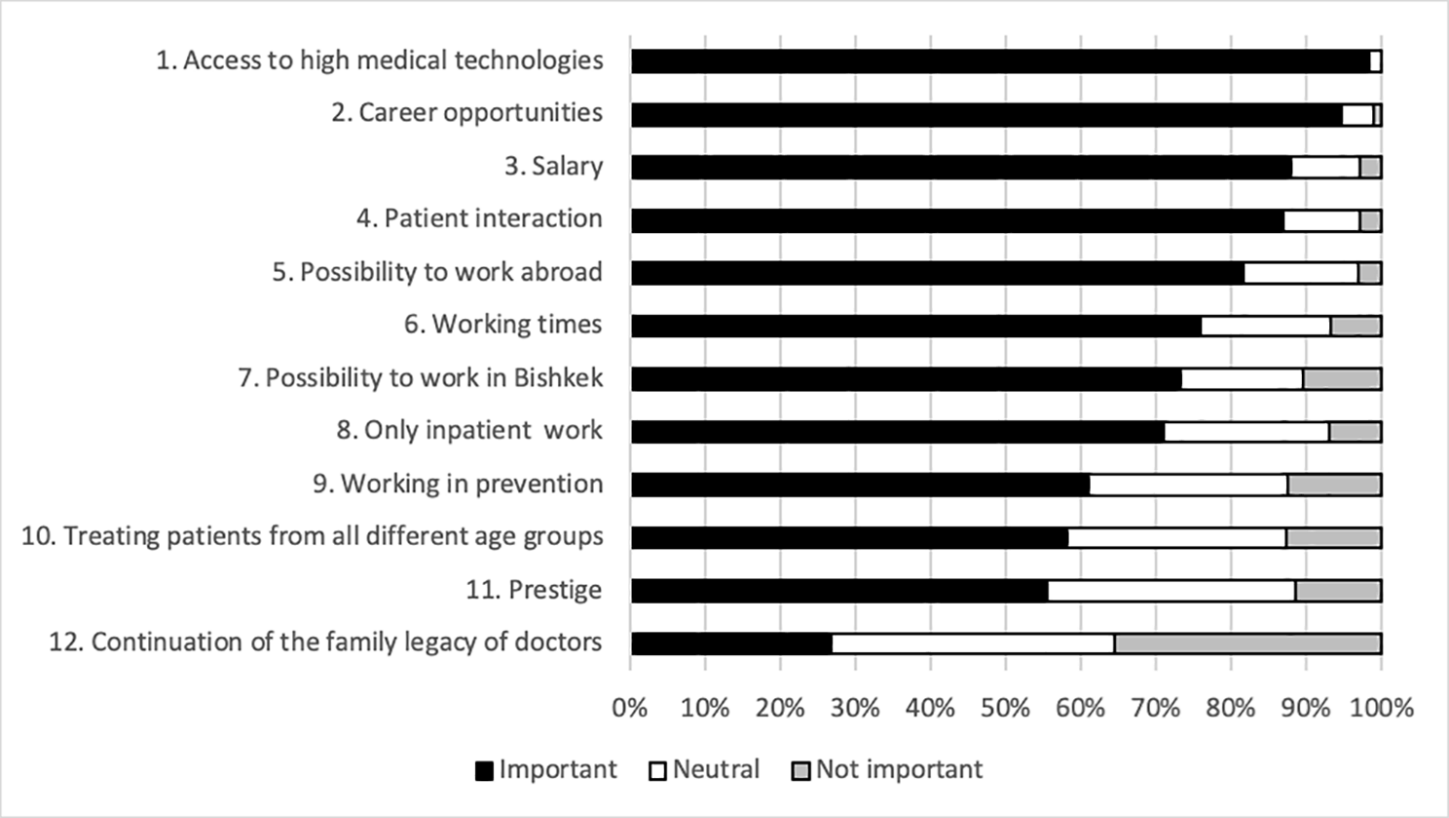
Ranking each of 12 factors from the most important to the least important for their specialty choice by Kyrgyz medical students Legend to table: N = 315 6th year students from KSMA surveyed in 2017.

Figure 4 presents the relative importance of 12 factors for the choice of specialty for 6th year students (N = 315.). Access to high medical technologies, career opportunities, salary, patient interaction and possibility to work abroad were the top 5 factors rated as important or very important by more than 80% of the students. The least important was the continuation of a family legacy of doctors (27%).

### d. Difficulty, attractiveness and prestige of FM compared to other specialties

Compared to the other 12 specialties FM was considered as very difficult by year 1 and year 6 students (Ranking in the pile sorting: 8 and 9.5/10). In contrast, its prestige and attractiveness, were considered moderate in year 1 (4.5 and 6.5/10 respectively) and were the lowest at year 6 (1.5/10 for both). In summary, year 6 students considered FM as the most difficult but less prestigious and attractive specialty.

### e. Students’ perception of FM

**Table** 3 presents the synthesis aggregating the quantitative (percentages by answers from the survey) and qualitative (quotes) findings organized around 7 themes presented by Olid et al.[26]. The key findings for each theme are presented below.

**Table 3:** Results from survey and focus groups A synthesis aggregates finding from both quantitative and qualitative findings.

THEMES	SURVEY				FOCUS GROUPS	SYNTHESIS BY THEME
	Items from survey	study year	scale		Quotes from focus groups	
			*agree*	*disagree*		
**Broad scope and context of practice**	It is difficult to become a good family doctor because it is such a wide field.	1	**65.18%**	7.04%	*"GP is the most difficult specialty, because of the great scope of work, similarly, neurosurgery, because it is difficult to regulate something in microsurgery, brain. Then goes traumatology. It is also difficult specialty. […]" (FG6-Y6)*	Students from the 3 study years considered GP as a specialty with wide field and a broad scope of practice, making it a difficult specialty. However, compared to year 1, more students in year 4 and 6 disagreed with the statement that GP provides most health care needed by patients .
		4	**66.03%**	13.86%		
		6	**68.88%**	12.06%		
	The family doctor is able to provide most of the health care patients require.	1	**43.33%**	19.63%	*"Family doctor is a very important profession as a family doctor. He provides treatment to everyone, irrespective of age, gender. Also a family doctor not only treats but helps his patients psychologicallygiving them consultations." (FG2 - Y1)*	
		4	**37.23%**	33.69%		
		6	**38.41%**	31.75%		
**Lower interest or intellectually less challenging**	In Kyrgyzstan working as a family doctor is not very attractive.	1	**37.04%**	29.26%	*“GP is boring. You must be a super unlucky fellow to be him.” (FG5-Y4)*	Most students from year 4 and 6 perceived FM as unattractive and with limited career possibilities. They were very critical towards the professional GP and repeatedly stated it was office work and boring. In their view, GP can only manage minor problems, and have to refer their patients to specialists. Year 6 students however modulate this view since about half of them do not agreee with the statement of referral to specialits. Year 1 students, although acknowledging that FM is unpopular and highlighting the lack of development perspective, had however a better image than older students (NB: about a third of year 1 students had a neutral answer to these statements).
		4	**66.30%**	13.59%		
		6	**69.84%**	8.25%		
	Family doctors have limited career possibilities.	1	**46.67%**	17.41%	*“For me, GP/FM is office work. You cannot become rich here; there is no career development.”(FG5-Y4)*	
		4	**71.20%**	10.87%		
		6	**75.87%**	8.89%		
	Family doctors are only able to manage minor health problems.	1	**37.78%**	28.52%	*“Why is GP/FM not attractive? Because family doctor has a lot of knowledge, but he cannot cure a patient completely, still he will have to send the patient to a specialist.” (FG4-Y1)*	
		4	**58.43%**	17.66%		
		6	**60.95%**	18.09%		
	When treating patients family doctors should at an early stage request additional support from a specialist.	1	**53.70%**	15.93%	*“From my experience, he [GP/FM] just directed me to a specialized doctor...” (FG4-Y1)*	
		4	**57.61%**	17.93%		
		6	**54.60%**	23.17%		
	The only task of the family doctor is to refer patients to the appropriate specialist.	1	34.45%	35.18%	*" Sometimes I think that GP just direct to a specialized doctor, since in the city a cardiologist doesn’t see patient without a referral from a specialized doctor. Many people just come and say: “My child is ill, give me a reference” and GP provides reference without examining a child... " (FG6-Y6)*	
		4	**42.66%**	35.87%		
		6	32.70%	**48.26%**		
	Access to specialists should be controlled and coordinated by family doctors.	1	**45.93%**	18.89%	*"Visiting a family doctor first must be mandatory." (FG2 Y 1)*	
		4	**52.99%**	24.73%		
		6	**55.87%**	21.27%		
**Influence of role models and society, other professionals and family**	The majority of teaching staff at university do not know what family medicine is about.	1	8.15%	**56.29%**	*"Training of the staff. They absolutely do not know who GPs are, they just say that we study according to the Bologna system and at the end of studying we will be GPs. But these are absolutely different things. Starting from the first year it is necessary to explain"(FG6-Y6)*	The focus groups revealed that the key positive influencers are the parents who practice family medicine and the rare professors who practice family medicine. Other professors and colleague on the contrary dismiss FM as shown in the survey. Students therefore only have rare role models within the society and family. students who learned about the family physician’s work at an early stage or whose close relative is a family physician demonstrated a better attitude and more interest in family medicine. Students reported that some teaching staff appeared not knowing what FM is about. Most importantly, most comments about FM and the reform made by professors, FD, students and alumni are negative.
		4	21.74%	**43.21%**		
		6	28.57%	**38.42%**		
	How were the comments about Family Medicine you heard while studying at KSMA?		***positive***	***negative***	*"Administration of the KSMA and our professors disrespectfully treat a new curriculum. We often hear their negative responses. They contributed to our negative perception of GP specialty". (FG6-Y6)*	
	Professors	4	13.58%	**42.67%**		
		6	17.14%	**47.62%**		
	Family physicians	4	16.57%	**39.94%**		
		6	21.27%	**44.13%**		
	Hospital physician	4	19.30%	**29.62%**		
		6	27.30%	**29.21%**		
	Students	4	7.07%	**68.21%**		
		6	6.34%	**78.41%**		
	Alumni	4	5.98%	**59.78%**		
		6	4.76%	**73.02%**		
**THEMES**	**SURVEY**				**FOCUS GROUPS**	**SYNTHESIS BY THEME**
	**Items from survey**	**study year**	**scale**		**Quotes from focus groups**	
			*agree*	*disagree*		
**Lower prestige**	Family doctors are poorly valued in our society.	1	32.97%	**35.19%**	*“We often hear: “He’s the son of this surgeon”, but never: “He’s the son of a family doctor.” (FG2-Y1)*	Students from the 3 study years recognized that FM is not prestigious. They perceived it as poorly valued by the society but also by other medical doctors. They considered that specialists are more needed than GP. However they also think that GP should be more prestigious. When evaluating prestige of the profession in comparison with other spialties in the focus group discussion, GP/FM profession ranked at the lowest. This low ranking was justified by the low salary this profession gets, the poor working condition, not recognized as a specialty and lack of professional development opportunities.
		4	**69.30%**	10.33%		
		6	**80.31%**	8.57%		
	Family doctors are poorly valued by other medical doctors.	1	27.03%	**35.92%**	*"As for GP – work is not seen. No one will remember the doctor".(FG5-Y4) * *« Honestly many people think that family doctors are not doctors » (FG2-Y1)*	
		4	**62.50%**	11.14%		
		6	**76.19%**	7.62%		
	In Kyrgyzstan narrow specialists are more needed than family doctors.	1	**44.44%**	20.37%	*“If a person wants to be a man of importance, hold a position specialty plays an important role. To be an oncologist is prestigious. To be a family doctor is not prestigious”. (FG3-Y6)*	
		4	**47.28%**	22.56%		
		6	**41.27%**	26.03%		
	A family doctor should have the same prestige as a specialist.	<	**52.59%**	10.37%	*"GP – great knowledge, it is difficult but not prestigious."(FG-Y6)* *“I agree with everyone. Profession of a family doctor is not popular now and it needs to be made competitive. In order to motivate students to become a family doctor, the officials have to provide good working and living conditions in order residents not to think how they will work, where they will live and what they will eat.” (FG1-Y4)*	
		4	**63.04%**	13.31%		
		6	**66.99%**	11.42%		
**Low remuneration**	Family doctors should receive a higher salary than narrow specialists.	1	26.29%	26.66%	*"Well, as for the question of what encouraged us to choose a carrier of a specialized doctor I can say that I was such a romantic freshman. I want to be this or that, maybe even a family doctor. But later I concluded that in the real world I must have a well-paid job to guarantee a comfortable life for my family and me. This is the main motive for choosing the specialty which can ensure a decent standard of living. Unfortunately I will get less satisfaction from this specialty than the one I really like." (FG3-Y6)*	The issue of remuneration is key in this profession and it is perceived by students of the 3 study years as much too low, not even allowing to live decently. It is thus a major obstacle to choosing FM Whereas year 1 students do not have a clear opinion about how GP should be payed (about 5 % are neutral with regard to the statement and the rest are shared between agreement and non-agreement), most year 4 and 6 students consider that GP should be better paid, even more than specialists.
		4	**48.37%**	14.40%		
		6	**63.49%**	15.24%		
**Medical school influences**	Everyone should receive training in family medicine, no matter what specialty he/she choose later.	1	**54.45%**	28.52%	*"We must know everything a family doctor does. Even if we are going to be specialized doctors, we should know everything. Even our relatives can ask us for medical help".(FG4-Y1)*	The opinion of the 1st year students that each student must complete a course in GP/FM at post-graduate level whatever specialty will be chosen testifies to their awareness and understanding of the current needs of the health care system. This stands in contrary to 4th and 6th students, who are less aware of FM reform. Generally, FM lectures are perceived as boring and this also linked to the teaching staff being unsufficiently informed and trained about the role of GPs says the students. The undergraduate training fails to stimulate interest of students towards FM. However year 4 students report some lectures who helped them to understand what FM is about.
		4	24.45%	**60.06%**		
		6	17.46%	**68.26%**		
	Lectures/trainings during yr1 have increased my interest in FM	4	9.24%	**60.59%**	*"When the lecture began we all complained that there was no reason to have this lecture. We couldn’t even imagine who a family doctor is, but after the lecture it became clear. It is especially important to communicate with a patient, not only give a prescription, but also discuss whether the patient can afford to buy the medicine. I realized that communication is sometimes more useful than medications".(FG4-Y1)*	
		6	7.62%	**68.89%**		
	Lectures/trainings during yr1 helped me to understand what FM and FD are	4	**45.11%**	26.36%		
		6	31.11%	**40.32%**		
	Lectures/trainings on general practice during yr5 have increased my interest towards FM	6	7.93%	**68.89%**	*"FM lessons are superficial, boring, not interesting. The teaching staff is not ready for teaching FM. Probably the problem is in the teaching staff who does not understand the work of GP. And we need optimism and patriotism". (FG6-Y6)*	
	The family medicine lectures/training during yr5 helped me to understand what family medicine is.	6	**42.54%**	30.16%		
	The lectures/trainings during yr5 gave me a comprehensive view on family medicine	6	30.15%	**40.00%**		
**Post-graduate training**	Two year residency prepares the students sufficiently for the most common medical situations they will encounter later.	6	**47.62%**	27.94%	*“Why should we waste two years?” (FG5-Y4) * *“I think this is not fair for non-government-subsidized students. We pay money, study for many years; we are made to have two years of FM residency. This is an impairment of students’ rights.”(FG1-Y4) * *“But here in Kyrgyzstan we pay for our residency training and get no money during this period.”(FG3-Y6)* *Core education takes six years plus two years of FM residency. In addition, one extra year of training if we want to become a specialized doctor”. (FG2-Y1) * *“What can we do? We will have 2 years FM residency training; it is not the end of the world. (FG6-Y6)”*	From the survey results, a majority of 6th year students agree that two years PGME is enough for FM and that it prepares students sufficiently. However, during the focus groups, they express great reluctance toward the 2-year postgraduate training in FM since the residency is unpaid and will be costly for their families. When discussing the post-grad training, students thought they would all have to become GP and disagreed with that. This misunderstanding may have caused their negative attitude towards the two-year residency training in family medicine. As for 1st year students, the focus group revealed that they are better informed of the ongoing reforms in the KR health care system and in medical education in particular, they have a more positive attitude to the post-graduate training in GP/FM and were fully aware about the 2 year post-grad training that would follow the 6 years of studies.
	Two year residency is enough for family medicine.	6	**48.25%**	31.74%

1. *Broad scope and context of practice*: Students from the 3 study years considered FM as a specialty with a wide field and a broad scope of practice, making it a difficult specialty.
2. *Lower interest or intellectually less challenging*: Most students from year 4 and 6 perceived FM as unattractive and with limited career possibilities. They were very critical towards the profession of FM and repeatedly stated it was office work and boring. In their view, FM doctors can only manage minor problems, and have to refer their patients to specialists. Year 1 students, although acknowledging that FM is unpopular and highlighted the lack of development perspective, had a better image than students in Year 4 and 6.
3. *Influence of role models and society, other professionals and family*: The key positive influencers are parents who practice FM and the rare professors who promote FM. Other professors on the contrary dismiss FM. Students therefore only have rare role models within society and family. Students reported that some teaching staff appeared not to know what FM is about. Worse, most comments about FM and the reform made by professors, family doctors, students and alumni were negative.
4. *Lower prestige*: Students from all study years recognized that FM is not prestigious. They perceived it as poorly valued by the society but also by other medical doctors. They considered that specialists are more needed than FM. However, they also think that FM should be more prestigious. The low prestige is justified by the low income this profession gets, the poor working condition, not recognized as a specialty and lack of professional development opportunities.
5. *Lower remuneration*: The issue of remuneration is indeed key in this profession and it is perceived by all students as much too low, not even allowing to live decently. It is thus a major obstacle to choosing FM.
6. *Medical school influences*: The positive opinion of the 1st year students on the completion of a course in FM at post-graduate level whatever specialty will be chosen is linked to their awareness and understanding of the current needs of the health care system. This stands in contrary to 4th and 6th year students, who are less aware of the reform. Generally, FM lectures are perceived as boring and this is also linked to the teaching staff being insufficiently informed and trained about the role of FM. Thus the undergraduate training fails to stimulate interest of students towards FM. However, year 4 students report some lectures which helped them to understand what FM is about.
7. *Post-graduate training*: From the survey results, a majority of 6th year students agree that two years PGME is enough for FM and that it prepares students sufficiently. However, during the focus groups, they express great reluctance toward the 2-year postgraduate training in FM since the residency is unpaid and will be costly for their families. When discussing the post-grad training, students thought they would all have to become FM doctors and disagreed with that. This misunderstanding may have caused their negative attitude towards the two-year residency training in FM . As for 1st year students, the focus group revealed that they were better informed of the ongoing reforms in the KR health care system and in medical education. In particular, they had a more positive attitude towards the post-graduate training in FM and were fully aware about the 2 year post-grad training that would follow the 6th years of studies.

In addition to these 7 themes, two additional themes emerged from the qualitative analysis of FGD that were not in the Olid et al.[26] framework. These themes could be specific to the Kyrgyz context.

### 1. Working conditions

This theme was defined as *Work location, salary and equipment in facilities allowing to practice in decent conditions.* Students expressed clearly their needs in terms of basic living and working conditions. In addition to the poor remuneration already discussed, they raised the issue of chronic lack of equipment in rural areas, which does not allow professionals to answer the needs of the population. They also raise the issue of language barrier, since Kyrgyz is not the first language taught at school whereas in rural areas the population mostly speaks Kyrgyz. These factors are highlighted through the two following quotes:

*“You have to understand, no matter how we study, no matter how we want to be a family doctor, if we would not be provided with basic working and living conditions, there won’t be a FM. If there is no first-aid room or medical treatment room, it will discourage residents to work there. Also, the students are afraid to stay in a small village or town because they won’t be able to provide their family.“ (FG1-Y4)*.

*“We need three factors. They are salary, working conditions, and equipment. It is true, even the building, and these walls treat patients.” (FG3-Y6)*.

### 2. Social accountability/responsibility

This theme was defined as the *Need of becoming FM as a mission with regard to the country needs*.

Some students consider that they have to help people in the remote areas, that it is important for the country. They also have to convince families and friends of the importance of this mission. This sense of duty to help the country was the strongest in year 1. This theme is described in the quotes from two students in Year 1 and another from Year 4.

*“If we tell our friends that the profession of a family doctor is awesome, they will follow us. For example, I called my parents and told them that the profession of FM is good and our generation can change the situation with this specialty in our country”.(FG2-Y1)*.

*“I think that is good. I am from Batken. I am government-sponsored student. Therefore, I am ready to go back to Batken. When I pass by hospitals, I see many people coming to Bishkek from the rural areas. Imagine coming from another town with their children to get medical help. Medical service is expensive and it takes long hours to get to the capital. However, if we go to rural areas, we will be able to help those people in their home town. (FG2-Y1)*

*“it is my childhood dream to become a doctor because my father and mother are doctors. Since childhood, I have always been amazed by their way of life and they have always inspired me. I can say that each doctor is a hero. I saw them getting up at night and going to a clinic or to the patient; they can refuse from family events and celebrations to help someone. Now I want to have the same experience.” (FG1-Y4)*.

## Discussion

### Main findings

Our results show that the interest of Kyrgyz students for FM was the lowest of all specialties particularly during the final study year. Access to high medical technologies, career opportunities, salary, patient interaction and possibility to work abroad were the most important factors influencing specialty choice. Using the framework by Olid et al. [26], the 7 themes were applicable to the Kyrgyz context and two additional themes emerged. FM is considered a difficult specialty due to its wide scope of work, but not attractive because treating only minor health problems and providing limited career possibilities. In addition, poor prestige and insufficient remuneration have a discouraging influence on the specialty choice, since decent living conditions are not guaranteed. The medical school itself has a negative influence on student perceptions, in particular through the detrimental comments that students hear about the FM profession. Moreover, the recent lengthening of the PGME training from 1 to 2 years, further dissuaded some residents to become FM doctors. The two additional themes that emerged were the deficient working conditions in rural areas in Kyrgyzstan and the social accountability and responsibility of becoming FM as a mission to meet the population’s needs. These additional themes specific to the Kyrgyz context, might also be relevant for other LMICs.

### Comparison with literature

One of the main problems revealed by our study was a distorted image of FM, which is a world-wide issue [16], as well as a career of low interest and prestige.[26] Family medicine is viewed as a monotonous and non-technological medical practice with no intellectual challenge.[16, 20, 21] Most students estimated that it had a lower status than hospital specialties and that the main aim of a FM doctor was to identify serious diseases/disorders in order to refer those patients for specialized care. [27, 28]

Our study further confirmed the prevalent influence of the medical school. The importance of the academic environment on the choice of career for medical students has been clearly documented. Two studies have shown that the curriculum and academic discourse can play significant role on student’s professional identification and specialty choice.[17, 18] Students from schools where FM specialty is disregarded were less likely to practice primary care [29, 30] and the academic discourse prevented students’ ability to identify with the practice of FM.[18] Moreover, an institutional culture not valuing FM through positive role models, and transmitting a distorted image of this specialty, for example through specialists’ negative attitudes towards family doctors were key features influencing student perceptions.[27, 29, 31–33].

Our results are aligned with other studies with regards to FM’s image, but provide additional insight that might be specific to the Kyrgyz context or LMICs in general. The poor perceptions of FM might be explained by the fact that the pregraduate training did not include a FM curriculum until recently, and that the post-graduate training lasted only one-year post-certification at the time of the study. Finally, students raised a discrepancy with their personal needs as defined by Querido [34] (salary, career options, status, work-life balance, labor content) and also illustrated by Fonkon et al. findings.[10] Indeed, as medical career decisions are formed by a matching of perceptions of specialty characteristics with personal needs [19, 34], it is easy to understand that working in remote rural areas, without the necessary equipment to diagnose and treat patients, and without a level of remuneration consistent with raising a family, were main obstacles to choosing this career. This is primarily a political issue out of the control of medical schools.

### Strengths and limitations

A key strength of this study is that it adds to the body of literature in central Asia. The framework used to analyse the findings did not include any literature from low-and-middle-income countries and our study thus complements what is already known. We also acknowledge some limitations. Because of timing and funding, this study was carried out as a cross-sectional rather than longitudinal approach. A longitudinal approach would have allowed to follow the cohort over a longer period of time and assess their changes in perception more precisely. Differences that are being observed between the different years of study could also be due to true differences in each cohort rather than changes that occur due to the course of the study. Furthermore, the cohort came from one single institution in Kyrgyzstan. However, KSMA is considered the biggest and, in the capital, including students from a variety of background as presented in the results. Whilst participation of students was voluntary the data collection happened during compulsory teaching hours. Students might therefore have not felt the freedom to leave or not answer the questionnaire. In addition, about one third of the students did not complete the survey and we cannot exclude that the perceptions about FM can be even worst in reality. Finally, we used an existing questionnaire that presents some weaknesses. It included some items that were formulated as leading questions: their wording may have favoured certain responses. The 5-point Likert scale for several questions allowed respondents to give differentiated opinions, however many chose not to express an opinion by selecting the neutral option.

## Conclusion - relevance for public health and medical education

This study highlighted the key factors responsible for the very low number of students choosing to become FM in Kyrgyzstan. FM is considered a difficult specialty due to its wide scope of work, but not attractive because treating only minor health problems and providing limited career possibilities. In addition, a major deterrent influence in this context is the poor working conditions encountered in remote areas, including lack of equipment and low remuneration, making it impossible to care properly for patients and live decently. This factor is presumably specific to many LMICs, it is out of reach of medical schools and has to be treated politically through improvements in the health system. Another prevalent influencer, common to many countries, is how medical schools through their institutional culture are not valuing FM through positive role models, and transmit a distorted image of this specialty. Successful interventions to increase the proportion of medical students choosing a FM career are characterized by diverse teaching formats, student selection, and good-quality teaching.[35] The most effective strategies consists in (a) developing longitudinal, multifaceted, FM programs during the medical curriculum, and provide a high-quality experience in PHC by introducing FM practice clerkships in pre- and postgraduate level; (b) general practice needs to be championed within the undergraduate curriculum[36], especially by building it as an academic discipline with academic FM doctors in prominent and senior roles both in teaching and research, and as a specialty on the same level than others. Having FM doctors as teachers in the curriculum will improve professional identity formation for the students.[17, 18].

These findings served as a basis for recommendations destined specifically to help Kyrgyzstan improve its health system. The package of measures seems to indicate there is an increase of residents choosing FM and going to the regions (2019) (Fig. 1). However, this aspect will have to be evaluated and studied in the long term.

## Electronic supplementary material

Below is the link to the electronic supplementary material.

Supplementary Material 1

## Data Availability

The datasets used and/or analysed during the current study are available from the corresponding author on reasonable request.

## Footnotes

1 Government subsidized students are called budget students in Kyrgyzstan.

2 **13 specialties**: Cardiologist, Dentist, Dermatologist, Family doctor, Gynaecologist, infectious disease specialist, Oncologist, Ophthalmologist, Neurologist, Neuro-surgeon, Psychiatrist, Surgeon, Traumatologist, Therapist (internal medicine), Urologist.
