# Supplementary material for: Exploring medical students’ perceptions of family medicine in Kyrgyzstan: a mixed method study
Source: BMC Med Educ. 2023 Apr 12;23:239. doi: 10.1186/s12909-023-04126-2 (PMC10099892; doi:10.1186/s12909-023-04126-2)
Supplement: Supplementary file 1 — Supplementary Material 1 [file 12909_2023_4126_MOESM1_ESM.pdf]

Perception of students on Family Medicine for 6-year students

Dear Student!

The research team of KSMA and the experts from the Geneva University Hospitals invite you to take part in a survey on the students' perception on family medicine. The results of this study are very important for further development of family medicine, as well as improvement of the curriculum at KSMA.

Your participation is entirely voluntary and anonymous.

The confidentiality of your answers is guaranteed. After reading the information sheet and signing the consent sheet; if you agree to participate:

- Carefully read the question
- Mark your correct answer
- Answer each question

Please take into account that at present, there is a variety of definitions for the concepts of “family medicine" and "general medical practice". Following the statement from the European Organization of Family Doctors (WONCA Europe), the terms "family medicine" and "general medical practice", as well as "family doctor" and "general practitioner" will be written either through the slash "/" or individually, implying that they are interchangeable.

In this survey we will use the terms “family medicine" and "family doctor".

MER
Swiss project

Demography

1. Sex

☐1 Male

☐2 Female

2. Age:………

3. Before starting your study at KSMA: Where did you live?

☐1 City ____

☐2 District center_______________________________

☐3 Rural area ____

☐4 Outside Kyrgyzstan

4. Year of medical study

☐1 1st ☐2 2nd ☐3 3rd ☐4 4th ☐5 5th ☐6 6th

5. What type of student are you?

☐1 Government-subsidized student (budget)

☐2 Contract student (private)

6. Have you ever consulted a family doctor?

☐1 no → please continue with **question 8**
☐2 yes →please continue with **question 7**

7. If yes, how satisfied have you been with your last visit to the family doctor?

☐1 very unsatisfied

☐2 unsatisfied

☐3 uncertain

☐4 satisfied

☐5 very satisfied

☐6 never consulted a family doctor

8. How did you first time hear about family medicine? (1 answer only)

☐1 TV/Radio/Newspapers

☐2 from university (e.g. teachings or administrative department)

☐3 remarks from teachers at school or university

☐4 from family members

☐5 from own experience with family doctor

☐6 other, please specify__________________________________

☐7 never consulted a family doctor

9. Is one of your close relatives (father, mother, brother, sister) a narrow specialist doctor?

☐1no
☐2yes

10. Is one of your close relatives (father, mother, brother, sister) a family doctor/general practitioner?

☐1no
☐2yes

Could you tick the box that fits best your opinion for each of the statements below?

Choice of specialty

11. How much are you interested in working in each of the following specialties/career options after your studies:

		Not interested at all	Not interested	Neutral	Interested	Very interested
11.1	Emergency Medicine	☐1	☐2	☐3	☐4	☐5
11.2	Family Medicine	☐1	☐2	☐3	☐4	☐5
11.3	Therapy	☐1	☐2	☐3	☐4	☐5
11.4	Obstetrics and Gynecology	☐1	☐2	☐3	☐4	☐5
11.5	Pediatrics	☐1	☐2	☐3	☐4	☐5
11.6	Psychiatry	☐1	☐2	☐3	☐4	☐5
11.7	Surgery	☐1	☐2	☐3	☐4	☐5
11.8	Other,please specify ____________________	☐1	☐2	☐3	☐4	☐5

12. What is the importance of the following aspects for your choice of specialty

		Not important at all	Not important	Neutral	Important	Very important
12.1	Salary	☐1	☐2	☐3	☐4	☐5
12.2	Prestige	☐1	☐2	☐3	☐4	☐5
12.3	Working times	☐1	☐2	☐3	☐4	☐5
12.4	Patient interaction	☐1	☐2	☐3	☐4	☐5
12.5	Treating patients from all different age groups	☐1	☐2	☐3	☐4	☐5
12.6	Working in prevention	☐1	☐2	☐3	☐4	☐5
12.7	Work in Hospital	☐1	☐2	☐3	☐4	☐5
12.8	Possibility to work in Bishkek	☐1	☐2	☐3	☐4	☐5
12.9	Possibility to work abroad	☐1	☐2	☐3	☐4	☐5
12.10	Access to high medical technologies	☐1	☐2	☐3	☐4	☐5
12.11	Career opportunities	☐1	☐2	☐3	☐4	☐5
12.12	Continuation of the family legacy of doctors	☐1	☐2	☐3	☐4	☐5

MER Swiss project

13. Do you agree or disagree with these general aspects/opinions of Family Medicine?

		Strongly Disagree	Disagree	Undecided	Agree	Strongly Agree
13.1	The majority of students do not know what family medicine is about.	☐1	☐2	☐3	☐4	☐5
13.2	The majority of teaching staff at university do not know what family medicine is about.	☐1	☐2	☐3	☐4	☐5
13.3	Family doctors are poorly valued in our society	☐1	☐2	☐3	☐4	☐5
13.4	Family doctors are poorly valued by other medical doctors	☐1	☐2	☐3	☐4	☐5
13.5	The family doctor is able to provide most of the health care patients require	☐1	☐2	☐3	☐4	☐5
13.6	In Kyrgyzstan working as a family doctor is not very attractive	☐1	☐2	☐3	☐4	☐5
13.7	Everyone should receive training in family medicine, no matter what specialty they choose later	☐1	☐2	☐3	☐4	☐5
13.8	The quality of care/treatment/services provided by family doctors is not so good.	☐1	☐2	☐3	☐4	☐5
13.9	Family doctors have limited career possibilities	☐1	☐2	☐3	☐4	☐5
13.10	Family doctors are only able to manage minor health problems	☐1	☐2	☐3	☐4	☐5
13.11	It is difficult to become a good family doctor because it is such a wide field.	☐1	☐2	☐3	☐4	☐5

14. Do you agree or disagree with these statements comparing family doctors with specialists

		Strongly Disagree	Disagree	Undecided	Agree	Strongly Agree
14.1	A family doctor should have the same prestige as a specialist	☐1	☐2	☐3	☐4	☐5
14.2	Family doctors should receive a higher salary as narrow specialists	☐1	☐2	☐3	☐4	☐5
14.3	When treating patients family doctors should at an early stage request additional support from a specialist	☐1	☐2	☐3	☐4	☐5
14.4	Access to specialists should be controlled and coordinated by family doctors	☐1	☐2	☐3	☐4	☐5
14.5	In Kyrgyzstan narrow specialists(e.g. surgeons) are more needed than family doctors	☐1	☐2	☐3	☐4	☐5
14.6	The only task of the family doctor is to refer patients to the appropriate specialist	☐1	☐2	☐3	☐4	☐5

MER Swiss project

Perception of family medicine during your studies at KSMA (For year 4 & 6)

15. How were the comments about Family Medicine while studying at KSMA?

		Very negative	Negative	Neutral	Positive	Very positive
15.1	Professors	☐1	☐2	☐3	☐4	☐5
15.2	Family physicians	☐1	☐2	☐3	☐4	☐5
15.3	Hospital physician	☐1	☐2	☐3	☐4	☐5
15.4	Students	☐1	☐2	☐3	☐4	☐5
15.5	Alumni	☐1	☐2	☐3	☐4	☐5

16. During the first year of medical training, you attended the lectures within the module "Human, society and health". Do you agree or disagree with the following statements about "Human, society and health" module?

		Strongly Disagree	Disagree	Undecided	Agree	Strongly Agree
16.1	Familymedicinelectures/trainings on HSHareare welldelivered	☐1	☐2	☐3	☐4	☐5
16.2	Theprofessorsarewellprepared	☐1	☐2	☐3	☐4	☐5
16.3	I learned new things at the lectures within HSH module	☐1	☐2	☐3	☐4	☐5
16.4	Lectures/trainings on HSH module helped me to understand what FM and FP are	☐1	☐2	☐3	☐4	☐5
16.5	Observation during patient consultations were very useful	☐1	☐2	☐3	☐4	☐5
16.6	Family medicine should be taught at every year during the studies	☐1	☐2	☐3	☐4	☐5
16.7	I have positive attitude towards family physician work after the lectures and trainings on HSH	☐1	☐2	☐3	☐4	☐5
16.8	Lectures/trainings on HSH module have increased my interest in FM	☐1	☐2	☐3	☐4	☐5
16.9	Lectures/trainings on HSH module will not help me in my future work assignments	☐1	☐2	☐3	☐4	☐5

MER Swiss project

If you are year 6, please continue with question 17 & 18. (Only for year 6)

17. You attended the series of lectures about family medicine during your 5th year, do you agree or disagree with the following statement?

		Strongly Disagree	Disagree	Undecided	Agree	Strongly Agree
17.1	Family medicine lectures/trainings are well delivered	☐1	☐2	☐3	☐4	☐5
17.2	The professors are well prepared	☐1	☐2	☐3	☐4	☐5
17.3	I learned new things during the trainings on FM	☐1	☐2	☐3	☐4	☐5
17.4	The family medicine lectures/training helped me to understand what family medicine is	☐1	☐2	☐3	☐4	☐5
17.5	The observations of doctors during patient consultations were very useful	☐1	☐2	☐3	☐4	☐5
17.6	The number of hours on FM is enough for in-depth familiarization with discipline	☐1	☐2	☐3	☐4	☐5
17.7	Lectures/trainings on general practice have increased my interest towards FM	☐1	☐2	☐3	☐4	☐5
17.8	The lectures/trainings gave me a comprehensive view on family medicine	☐1	☐2	☐3	☐4	☐5
17.9	The lectures/training on family medicine will not help me for my future job assignments	☐1	☐2	☐3	☐4	☐5

18. Do you agree or disagree with the following statements about the post-graduate specialization training

		Strongly Disagree	Disagree	Undecided	Agree	Strongly Agree
18.1	Two year residency in general practice prepares the residents sufficiently for the most common medical situations	☐1	☐2	☐3	☐4	☐5
18.2	two-year residency is enough for family medicine	☐1	☐2	☐3	☐4	☐5
18.3	Some specialties should havea residency period of more than two years	☐1	☐2	☐3	☐4	☐5

Dear student! The second phase of the study will involve some interview within the focus groups. Please, if you are willing to participate in the focus group, choose “Yes” or “No”.

Many thanks for your participation!
